# The Complex Role of Infectious Agents in Human Cutaneous T-Cell Lymphoma Pathogenesis: From Candidate Etiological Factors to Potential Therapeutics

**DOI:** 10.3390/pathogens13030184

**Published:** 2024-02-20

**Authors:** Assia Angelova, Jean Rommelaere, Guy Ungerechts

**Affiliations:** 1 Clinical Cooperation Unit Virotherapy, German Cancer Research Center (DKFZ), 69120 Heidelberg, Germany; j.rommelaere@dkfz-heidelberg.de (J.R.); guy.ungerechts@nct-heidelberg.de (G.U.); 2 Department of Medical Oncology, National Center for Tumor Diseases Heidelberg and Heidelberg University Hospital, 69120 Heidelberg, Germany

**Keywords:** cutaneous T-cell lymphoma, infectious etiology, *Parvoviridae*, cutavirus, oncolytic H-1 parvovirus

## Abstract

Cutaneous T-cell lymphoma (CTCL) is a devastating, potentially fatal T-lymphocyte malignancy affecting the skin. Despite all efforts, the etiology of this disease remains unknown. Infectious agents have long been suspected as factors or co-factors in CTCL pathogenesis. This review deals with the panel of bacterial and viral pathogens that have been investigated so far in an attempt to establish a potential link between infection/carriage and CTCL development. A special focus is given to a recently discovered human protoparvovirus, namely the cutavirus (CutaV), which has emerged as a plausible CTCL etiological agent. Available evidence in support of this hypothesis as well as alternative interpretations and uncertainties raised by some conflicting data are discussed. The complexity and multifacetedness of the *Parvoviridae* family of viruses are illustrated by presenting another protoparvovirus, the rat H-1 parvovirus (H-1PV). H-1PV belongs to the same genus as the CutaV but carries considerable potential for therapeutic applications in cutaneous lymphoma.

## 1. Introduction

Cutaneous T-cell lymphoma (CTCL) belongs to the heterogeneous group of primary cutaneous lymphomas (CLs), the second most common extranodal non-Hodgkin hematological malignancy. CTCL accounts for approximately 75% of all CLs [1]. This disease emerges through the clonal proliferation of CD4+CD8- T lymphocytes that home to the skin. The two major CTCL subtypes are mycosis fungoides (MF), characterized by the formation of patches, plaques, and skin tumors, and Sézary syndrome (SS) with typical leukemic involvement and a generally poor prognosis [2]. The incidence of CTCL increases with age. CTCL patients are likely to develop various secondary malignancies such as other non-Hodgkin lymphomas, melanomas, and lung or bladder cancer [3].

It has been suggested that antigen-driven T-cell proliferation induced by medication use [4], genetic predisposition [5], or somatic mutations in signaling pathways [6] may contribute to CTCL pathogenesis. However, controversial data have been obtained, and the etiology of the disease remains unknown. Going back to the 1990s, Whittemore et al. were the first to raise the hypothesis that various infectious agents might be involved in CTCL development [7]. The current review summarizes the current knowledge on the infectious etiology of CTCL and focuses in particular on the still putative role of some parvoviruses in the induction of CTCL carcinogenesis. A recently discovered intriguing property of other parvoviruses, namely to exert, in contrast, therapeutic effects in CTCL, is also discussed.

## 2. Infectious Agents Involved in the Etiopathogenesis of Cutaneous Lymphoma

Infectious agents are known to induce cancers by acting in either direct or indirect ways. Direct carcinogenesis is exerted, e.g., by the oncogenic viruses (papillomaviruses, polyomaviruses, retroviruses, and herpesviruses, among others), which initiate infections leading, through direct virus-driven mechanisms, to malignant cell transformations. Indirect carcinogenesis is typically associated with chronic infections and inflammation. In CTCL, the malignant T-cell population consists of various clones that share a common TCR-Vß epitope, in contrast to the malignant T-lymphocyte clonal expansion characteristic of other lymphomas [8]. Since the ability to initiate polyclonal T-cell expansion in a Vß-restricted manner is characteristic of pathogen-produced immunostimulatory molecules known as superantigens, it was proposed that in CTCL carcinogenesis a bacterial and/or viral superantigen might serve as the trigger of chronic antigen stimulation and excessive T-cell proliferation.

### 2.1. Bacterial Superantigens in CTCL

#### 2.1.1. *Staphylococcus aureus*

A primary role of *S. aureus* in triggering CTCL carcinogenesis has not been proven so far. However, an association of CTCL clinical behavior with *S. aureus* colonization was noted. A trend towards higher antibiotic-sensitive *S. aureus* colonization was observed in CTCL patients in comparison with healthy control subjects, and colonization was suggested to be directly related to the body surface area of CTCL involvement [9]. Furthermore, a relationship between *S. aureus*-induced sepsis and lymphoma progression was found [10]. Conversely, treatment of infection with antibiotics was reported to lead to significant clinical improvement in *S. aureus*-colonized MF patients [11,12]. Based on the above, it was initially suggested that staphylococcal superantigens might be strongly and directly involved in CTCL pathogenesis. Indeed, the *S. aureus*-derived superantigen family of proteins is known to consist of at least 26 different paralogues that exert their activities by crosslinking the major histocompatibility complex (MHC) type II molecules with TCR-Vß, resulting in aberrant T-cell proliferation and proinflammatory cytokine release [13]. However, subsequent in vitro studies revealed that *S. aureus*-derived superantigens induce superantigen-responsive non-malignant T-cell proliferation rather than direct CTCL cell growth stimulation [14]. Staphylococcal infection-associated tumor cell expansion in CTCL therefore seems to rely on crosstalk between malignant and non-malignant T lymphocytes against the background of *S. aureus* superantigen-induced chronic antigen stimulation.

#### 2.1.2. *Borrelia burgdorferi*

*B. burgdorferi* is another bacterial agent that was suspected to play a plausible role in CTCL etiology. *B. burgdorferi*-specific flagellin gene sequences were detected in approximately 18% of the MF cases registered in a Lyme disease-endemic area in northeastern Italy but were not detected in any of the healthy control biopsies [15]. This bacterial pathogen is known to affect the complement function by inactivating complement regulatory proteins [16]. Moreover, *B. burgdorferi* is capable of persisting at immunoprivileged sites [17] using various immune evasion mechanisms, including outer-membrane antigenic variations [18]. It was suggested that *B. burgdorferi*, in particular in Lyme disease-endemic areas where long-term persistence-prone strains prevail, might function as a co-factor in MF pathogenesis and lead, through long-lasting antigen stimulation, to the malignant accumulation of skin-homing CD4+ lymphocytes. However, while improvement after *B. burgdorferi*-directed specific antibiotic therapy was described in other lymphoma types [19], the causal link between *B. burgdorferi* and MF remains controversial.

#### 2.1.3. *Chlamydia pneumoniae*

Abrams et al. speculated that there might be an association between *C. pneumoniae* infection and CTCL development. The authors identified an effector molecule originally designated as Sézary T cell-activating factor (SAF). SAF was defined as an inducer of interleukin-2 (IL-2) receptors on both normal and malignant T cells from SS patients. Despite the fact that SAF was produced by mitogen-stimulated peripheral blood mononuclear cells (PBMCs) from these patients, the authors failed to isolate the SAF-encoding gene from eukaryotic libraries. Since SAF activity was detected in the cytoplasm of SS-derived malignant cells, together with RNA-DNA complexes, it was suggested that this molecule was not of eukaryotic origin but was derived from a cytoplasmically replicating intracellular pathogen [20,21]. Indeed, using a panel of antichlamydial antibodies, immunoelectron microscopy, and Western blotting, the authors demonstrated that the majority of MF patients were positive for *Chlamydia* determinants. Furthermore, *C. pneumoniae* DNA was detected in skin and lymph node samples from MF and SS patients, respectively [21]. Interestingly, it was also shown that this bacterium might chronically infect normal keratinocytes, thus leading to the expansion of *C. pneumoniae*-specific skin-homing T lymphocytes and CTCL development. However, it has not been elucidated whether SAF is physically associated with the bacterium or produced during its life cycle. It cannot be excluded that SAF may be a eukaryotic product that tends to localize within bacterial inclusions. *C. pneumoniae*’s involvement in the etiopathogenesis of CTCL therefore remains controversial and needs further investigation.

### 2.2. Viral Superantigens in CTCL

In addition to the bacterial agents presented above, several viruses characterized by their ability to provide chronic immune stimulation have been studied for their potential involvement in CTCL development.

#### 2.2.1. Retroviruses: Human T-Lymphotropic Virus (HTLV)

In the 1990s, the hypothesis arose that retroviral infection of skin- and lymph node-resident Langerhans cells might be the triggering event in CTCL development [22]. Retrovirus-like particles were indeed found in these cells [23], and in 1980, the first publication was released describing the detection and isolation of the causative agent of human adult T-cell leukemia and lymphoma (ATL), HTLV, from MF patient’s PBMCs [24]. The HTLV etiology hypothesis was further strengthened by well-established significant clinical and histopathological similarities between CTCL and ATL and by several later discoveries. HTLV-specific sequences and reverse transcriptase activity were detected in a cell line derived from an SS patient [25] and in cultured PBMCs from CTCL patients [26,27]. In contrast to the above reports, which speak in favor of HTLV association with CTCL pathogenesis, other studies failed to amplify HTLV-specific sequences from skin lesion- and PBMC-derived CTCL patient DNA [28,29,30,31]. In one 2010 study, Bonin et al. analyzed 83 skin biopsies from MF patients in comparison with 83 healthy subjects’ skin samples. HTLV-I-like genomic sequences were found in 41% of the MF cases. Surprisingly, the frequency of HTLV-I detection in the control biopsies was also substantial, raising doubt about the direct role of this virus in CTCL carcinogenesis [32].

#### 2.2.2. Herpesviruses: Epstein–Barr Virus (EBV) and Cytomegalovirus (CMV)

These viruses are known for their characteristic capacity for long-term persistence through the establishment of latent infections. It was therefore speculated that both EBV and CMV might readily exert chronic antigen stimulation, leading to T-cell hyperproliferation. Indeed, EBV and CMV genomic sequences and seropositivity were reported to be present in CTCL samples by various studies [33,34,35,36]. However, these data could not be reproduced by other groups who failed to trace a link between either EBV or CMV and CTCL [37,38]. Similar inconsistencies accompanied attempts to link the Kaposi sarcoma-associated herpesvirus (KSHV or HHV-8) and human herpesviruses 6 and 7 (HHV-6 and HHV-7) to CTCL. While some groups could prove the prevalence of KSHV infection and the presence of virus-specific DNA in CTCL patient samples [39], others either observed a uniform negativity [34,40] or failed to immunohistochemically confirm polymerase chain reaction (PCR)-detected virus DNA positivity [37]. Only a small percentage, if any, of the tested samples were shown to be positive for HHV-6 or HHV-7 DNA [33,34,41,42], thus failing to support any significant role of these herpesviruses in CTCL pathogenesis.

Other viruses, namely the Merkel polyomavirus, which affects the skin and possibly causes Merkel cell carcinoma, were also suggested as CTCL-triggering/associated agents. The link between this virus and CTCL was nevertheless unquestionably rejected [43]. 

In this way, none of the bacterial or viral agents discussed above could show a convincingly consistent association with CTCL. Therefore, the infectious etiology of CTCL remained a matter of speculation for decades, until recently, when a novel viral candidate was discovered in MF specimens (see below). Infectious agents of both bacterial and viral origin that have been associated or investigated for an association with CTCL so far are listed in Table 1.

## 3. Parvoviruses Associated with the Etiopathogenesis of Cutaneous Lymphoma

### 3.1. The Parvoviridae Family

*Parvoviridae* is a large and heterogeneous family of single-stranded DNA viruses that can infect both invertebrates (subfamily *Densovirinae*) and vertebrates (subfamily *Parvovirinae*). The linear genome (about 3.9–6 kb) is protected by a non-enveloped icosahedral capsid with a diameter of approximately 26 nm. The parvoviral genome is divided into two major open reading frames (ORFs) that encode nonstructural (NS) and structural viral proteins (VPs). The coding region of the parvoviral genome is flanked by terminal repeats folded into hairpin-like structures that can be either identical or not [52,53]. The parvoviral major nonstructural protein, NS1, is strongly involved in virus replication and pathogenicity through its broad-spectrum enzymatic and sequence-specific DNA binding activities [54,55]. Parvoviruses are ancient viruses: it is believed that they have been circulating in nature for 90 million years [56,57] and acquiring various strategies for host adaptation and immune evasion. Parvoviruses are broadly distributed in nature: various animal hosts can be infected simultaneously by more than one parvovirus [58]. Most parvoviruses produce little or no disease in their hosts. However, some family members, in particular the canine parvovirus (CPV) [59] and the human parvovirus B19 [60], can cause severe clinical illness. For over three decades, the B19 parvovirus was the only known parvoviral human pathogen. This limitation was partly due to difficulties in propagating parvovirus virions in cell cultures. However, in recent years numerous new techniques and approaches have been introduced into the laboratory routine, which led to the discovery of novel parvoviruses and the introduction of new genera and species to the *Parvovirinae* subfamily [61] (Table 2). The paragraphs below focus on recently discovered human parvoviruses named cutaviruses in the context of their possible association with cutaneous lymphoma.

### 3.2. Cutavirus Discovery

In 2016, Phan et al. described the genome of a new protoparvovirus (Table 2) that was detected through metagenomics analysis and nested PCR in human diarrhea samples and MF biopsies [44]. Seven genomic sequences were identified in fecal samples from children in Brazil and Botswana. The viruses were named cutaviruses (CutaVs) and were found to share significant amino acid identity with bufaviruses, another recently discovered species belonging to the *Protoparvovirus* genus [45]. The study by Phan et al. analyzed a limited number of CTCL samples (17 in total), some of which (n = 2) were processed for virion-associated nucleic acid enrichment. These data did not allow the researchers to determine whether CutaV DNA detection was merely associated with ongoing replication or virion deposition in the skin or if it reflected a direct oncogenic role of the virus in CTCL. Furthermore, CutaV expression could not be convincingly shown in the transformed T lymphocytes infiltrating the MF-affected skin. 

### 3.3. Cutaviruses Association with CTCL

While no direct pathogenic role of CutaVs in CTCL could be concluded based on the above study, a later work by Väisänen et al. suggested that dermal CutaV DNA carriage might be strongly associated with cutaneous lymphoma. The authors set up multiplex quantitative PCR to analyze malignant and non-malignant skin samples from 25 CTCL patients as well as 98 healthy control subjects. CutaV DNA was detected in 16% (4/25) of the biopsies examined but, importantly, none of the healthy control samples. Interestingly, positive samples were derived not only from MF patients but also from an SS patient [46]. The association of CutaVs with CTCL was further demonstrated by another study that was launched to retrospectively analyze a large number of lesional skin samples from patients with CTCL. A total of 170 CTCL tissue specimens from 117 CTCL patients were analyzed by real-time PCR. CutaV DNA was found in 6/170 CTCL biopsies (taken from 6 of the 130 CTCL patients studied). Interestingly, none of the additionally investigated samples derived from cutaneous B-cell lymphoma patients displayed CutaV DNA positivity [47].

Large plaque-type parapsoriasis is considered to be a premalignant condition capable of developing into CTCL [48]. While Phan et al. failed to detect CutaV DNA in parapsoriasis samples [44], Mohanraj et al., in contrast, observed a significant association of CutaV carriage with plaque-type parapsoriasis. In contrast to the infrequent detection of other parvoviruses or other virus types (herpes or polyoma), CutaV DNA was consistently found in skin biopsies and skin swabs from parapsoriasis patients but was only occasionally found (1.96%) in healthy subjects, specifically in a skin swab from a single healthy individual. Notably, half of the patients who carried CutaV DNA in their skin subsequently developed CTCL. The latter findings suggested the idea of using CutaV DNA carriage as a potential biomarker for cutaneous lymphoma development [49]. 

The observations presented above hinted at a possible association of CutaV infection/carriage with CTCL (etio)pathogenesis. However, some recently published studies produced conflicting results. While Hashida et al. found a significant association of CutaV load with MF in a subset of Japanese patients [50], Bergallo et al. failed to detect the virus in 55 Italian patients’ skin specimens [51]. The current state of the art of research exploring the association of CutaVs with CTCL is summarized in Table 3. It shows that further investigations are obviously needed in order to reveal a straightforward link between CutaVs and CTCL and to elucidate whether these protoparvoviruses are a factor, a co-factor, or a satellite in cutaneous lymphoma. As discussed below, parvoviruses are endowed with oncotropic properties, implying that their association with neoplastic tissues may be opportunistic rather than causative.

## 4. Parvoviruses with Therapeutic Potential in Cutaneous Lymphoma

### 4.1. The Rat H-1 Parvovirus

The rat H-1 parvovirus (H-1PV) is another member of the *Protoparvovirus* genus (Table 2). H-1PVwas discovered in the late 1950s in transplantable human tumor samples and was initially thought to be an oncogenic virus [62]. However, further observations revealed that virus infection was not an initiating event in carcinogenesis but rather an opportunistic process based on the natural tropism of H-1PV for human cancer cells [63]. It was subsequently shown that infection with this protoparvovirus may lead to the suppression of both virally or chemically induced and spontaneously developed tumors in animal models [64,65]. This groundbreaking research inspired the idea that H-1PV may be successfully used as a selective cancer-killing agent and laid the groundwork for oncolytic parvovirotherapy. Numerous later investigations into the potential as a cancer therapeutic of H-1PV demonstrated that this virus possesses a broad range of anticancer activities against various types of cancers both in vitro and in animal models [66,67,68]. Moreover, it was shown that H-1PV-induced death of tumor cells may be immunogenic and may trigger the mounting of an anticancer immune response [69,70]. In 2011, H-1PV became the first parvovirus to be the subject of a bona fide clinical trial. The trial involved patients with progressive recurrent glioblastoma who were treated with either local (intratumoral/intracerebral) or systemic (intravenous) virus applications. The study demonstrated that H-1PV had excellent safety and tolerability in both applications. Furthermore, it provided the first hints of H-1PV treatment-associated immune system stimulation and warming of the tumor microenvironment [71].The outstanding tolerability of H-1PV, with no dose-limiting toxicities, was confirmed in a subsequent phase II trial in inoperable metastatic pancreatic cancer patients. Following virus administration, partial responses associated with favorable immune modulation were observed in two out of seven patients that were enrolled [72]. Altogether, over a century of H-1PV research has convincingly demonstrated that this oncolytic parvovirus deserves to be considered for clinical translation as a cancer immunotherapeutic.

### 4.2. The Potential of H-1PV against Cutaneous Lymphoma

H-1PV therapeutic potential in some hematological malignancies has already been shown. In particular, it was demonstrated that H-1PV is able to cause B-cell lymphoma regression and survival prolongation in animal models of Burkitt’s lymphoma (BL). Importantly, treating actively dividing normal B lymphocytes from healthy donors with H-1PV resulted in minor cytotoxicity levels, which were not related to virus gene expression. Normal memory (CD19^+^CD27^+^) B cells, the closest counterpart of malignant BL cells, were similarly resistant to H-1PV infection [73]. H-1PV infection of other types of blood cancer cells (diffuse large B-cell lymphoma and large-cell immunoblastic lymphoma, among others) similarly resulted in tumor cell death and reductions in cancer culture viability [74]. Remarkably, H-1PV was capable of suppressing the proliferation not only of malignant B lymphocytes but also T lymphocytes. Oncolysis was observed in T-cell acute lymphoblastic leukemia (T-ALL) and, of note, in CTCL cells [74]. H-1PV infection of SS-derived T-lymphoblast HH cells was characterized by efficient virus uptake, intracellular expression and replication, and ultimately host cell death with the release of infectious virion progeny [74]. Further studies are required in order to substantiate the potential of H-1PV in CTCL treatment. However, the first in vitro observations are promising and promote parvovirotherapy as a potential novel approach in cutaneous lymphoma. Thus, two *Parvovirinae* subfamily members, CutaV and H-1PV, belonging to the same genus (*Protoparvovirus*) seem to have distinct relationships with CTCL development. The pro-curative (oncolytic) effect of H-1PVon CTCL cells challenges the association of CutaVs with CTCL to be causative as opposed to opportunistic.

## 5. Conclusions and Future Directions

Despite all efforts to identify an infectious agent of either bacterial or viral origin that might be involved in CTCL development, the trigger of this malignancy remains unclear. The recently discovered human cutaviruses emerged as sound candidates with etiopathogenic roles in this disease. However, existing study limitations and controversial results hint at the need to await further investigations in order to draw a straightforward conclusion. Further studies are similarly required and worth considering to unravel the therapeutic potential of the oncolytic H-1 parvovirus in cutaneous lymphoma.

## Figures and Tables

**Table 1:** Infectious agents investigated for their association with CTCL pathogenesis.

Origin	Infectious Agent	Taxonomical Classification (Family)	References
	*Staphylococcus aureus*	*Staphylococcaceae*	[9,10,11,12,13,14]
**Bacterial**	*Borrelia burgdorferi*	*Borreliaceae*	[15,16,17,18,19]
	*Chlamydia pneumoniae*	*Chlamydiaceae*	[20,21]
	Human T-lymphotropic virus (HTLV)	*Retroviridae*	[22,23,24,25,26,27,28,29,30,31,32]
	Epstein–Barr virus (EBV)	*Orthoherpesviridae*	[33,34,35,36,37,38]
**Viral**	Human cytomegalovirus (CMV)	*Orthoherpesviridae*	[33,34,35,36,37,38]
	Kaposi sarcoma-associated herpesvirus (KSHV)	*Orthoherpesviridae*	[34,39,40]
	Human herpesviruses 6 and 7 (HHV-6 and HHV-7)	*Orthoherpesviridae*	[33,34,41,42]
	Merkel polyomavirus	*Polyomaviridae*	[43]
	**Cutavirus (CutaV)**	***Parvoviridae***	[44,45,46,47,48,49,50,51]

**Table 2:** Current International Committee on Taxonomy of Viruses (ICTV) classification of the *Parvoviridae* family.

Family	*Parvoviridae*		
**Subfamily**	*Densovirinae*	*Hamaparvovirinae*	***Parvovirinae***
**Genera (number)**	11	5	***Protoparvovirus*** *
			+10
**Species (number)**	37	37	***Protoparvovirus primate3*** (cutavirus)
			***Protoparvovirus rodent1*** (H-1 parvovirus)
			+89

* The heterogeneous *Protoparvovirus* genus incorporates 91 virus species, two of which (the cutavirus and the rodent H-1 parvovirus) play distinct roles (pathogenesis-associated and oncolytic roles, respectively) in cutaneous lymphoma (from Current ICTV Taxonomy Release|ICTV, https://ictv.global/taxonomy; accessed on 17 November 2023).

**Table 3:** Studies investigating the association of CutaVs with CTCL.

Study	Study Limitation(s)	Outcome	Conclusion
[54]	Limited sample Samples processed for virion nucleic acid enrichment	The genome of CutaV described for the first time. CutaV expression in tumor cells not detected.	No direct oncogenic role of CutaV in CTCL could be concluded.
[56]	Limited sample	CutaV DNA detected in MF- and SS-derived samples. CutaV DNA not detected in healthy control samples.	Dermal CutaV DNA carriage may be strongly associated with CTCL.
[57]	No healthy controls included	CutaV DNA detected in 6/170 CTCL (MF) biopsies. CutaV DNA not found in B cell-type cutaneous lymphoma samples.	CutaV DNA carriage may be associated with MF.
[59]	Limited sample	CutaV DNA consistently found in CTCL precursor lesion (parapsoriasis) samples. CutaV DNA only occasionally (<2%) detected in healthy control samples. In total, 50% of the CutaV DNA-positive parapsoriasis patients subsequently developed CTCL.	CutaV DNA carriage may serve as a predictive biomarker for CTCL development.
[60]	No healthy controls included	Frequent detection of CutaV DNA in MF patient samples (relative to other CTCL subtypes). CutaV positivity associated with shorter MF patient survival.	CutaV DNA carriage may be associated with MF.
[61]	No healthy controls included	CutaV DNA detected in 0/55 CTCL samples.	CutaV’s association with CTCL could not be demonstrated.

Abbreviations: CutaV, cutavirus; MF, mycosis fungoides; SS, Sézary syndrome.

## Data Availability

Not applicable.
